# The Fifty Year Rehabilitation of the Egg

**DOI:** 10.3390/nu7105429

**Published:** 2015-10-21

**Authors:** Donald J. McNamara

**Affiliations:** Eggs for Health Consulting, 5905 Cozumel Pl., Las Vegas, NV 89131, USA; djmmcnamara@gmail.com; Tel.: +1-202-550-7973

**Keywords:** eggs, dietary cholesterol, plasma cholesterol, lipoproteins, choline, xanthophylls

## Abstract

The 1968 American Heart Association announced a dietary recommendation that all individuals consume less than 300 mg of dietary cholesterol per day and no more than three whole eggs per week. This recommendation has not only significantly impacted the dietary patterns of the population, but also resulted in the public limiting a highly nutritious and affordable source of high quality nutrients, including choline which was limited in the diets of most individuals. The egg industry addressed the egg issue with research documenting the minimal effect of egg intake on plasma lipoprotein levels, as well as research verifying the importance of egg nutrients in a variety of issues related to health promotion. In 2015 dietary cholesterol and egg restrictions have been dropped by most health promotion agencies worldwide and recommended to be dropped from the 2015 Dietary Guidelines for Americans.

## 1. Introduction

Of the vast variety of foods that humans consume, only one has ever been specifically singled out for restriction in an effort to reduce cardiovascular disease (CVD) risk in the population—the egg. The most widely known dietary recommendation in the world is the 1968 admonition from the American Heart Association (AHA) to consume no more than three egg yolks per week [1]. For consumers this provided a recommendation they could easily incorporate into their lifestyles (in 1968 who knew what 10% of calories from saturated fat or 300 mg cholesterol actually meant); for health professionals it was easy to explain dietary equivalency (high dietary cholesterol equals high blood cholesterol equals high CVD risk) without getting into detailed explanations of fat, calories, *etc.*; and for the media it was an ideal icon for the hazards of our modern dietary patterns which lead to high CVD incidence. The only negative effect was on the egg industry which saw a substantial drop in per capita egg consumption over the subsequent years. The question many scientists raised was whether or not this recommendation would actually have any impact on CVD rates. But like the mistaken views in nutritional sciences, it was thought that it couldn’t hurt. In the long run it turned out that not only was the recommendation based on misunderstood data and effectively useless, it actually did damage to the general public in terms of their nutritional needs.

## 2. The Egg Industry Responds

The egg industry was faced with a difficult situation in 1968: fight back and be accused of putting profits ahead of public health or simply give in to the dietary cholesterol phobia being promoted by the AHA and later on by the US government. The other challenge, should the industry decide to finance research in an attempt to prove the egg innocent of the charges, was the fact that in science the accusation that the findings of any study from “industry funded research” are often a quick nullification of the credibility of the results and the investigators involved. But the attacks from health promotion organizations and the media on how harmful meat, dairy and eggs were because of their fat and cholesterol content necessitated that the animal food commodity groups respond, and overall their responses were based on the use of science. The decline in egg consumption in the US after the AHA recommendations were publicized, and further still after the 26 March 1984 Time magazine cover (which referred to a drug study), prompted the US egg industry to establish the Egg Nutrition Center (ENC) to promote research and initiate health education efforts to address issues raised by the AHA in 1968, the Select Committee on Nutrition in 1977 (predecessor of the Dietary Guidelines Advisory Committee), and eventually the National Cholesterol Education Program (NCEP) of the Heart, Lung and Blood Institute of the National Institutes of Health (NIH). In addition, a number of non-government organizations (NGOs) had gotten on the bad egg, bad cholesterol bandwagon as an effective way to raise funds and there was a virtual avalanche of new food products touting “low-cholesterol” and “cholesterol-free” (cholesterol-free peanut butter?) in print, radio and TV advertising. And for most people the concept of “low cholesterol” was a confusion between dietary cholesterol and blood cholesterol. The evils of cholesterol, and eggs, were constantly presented to the consumer.

The egg industry’s goal in forming ENC was to formulate a scientific basis for addressing the dietary cholesterol issue. ENC formed a scientific advisory panel in 1984 composed of clinical and university research scientists to help formulate a long range research plan, as well as to serve as consultants to the industry and spokespersons on behalf of the industry. Contrary to the “consensus” argument, not all nutritional scientists were convinced that the low-fat, low-cholesterol diet was the best answer to our CVD problems. I served on that original scientific advisory panel because I had carried out studies on the effects of egg intake on blood cholesterol levels and endogenous cholesterol metabolism [2] and had serious doubts regarding the contribution of dietary cholesterol to blood cholesterol levels and CVD risk [3].

The scientific bases for the original dietary cholesterol restriction was based on three lines of evidence: animal studies showing cholesterol in the diet increased plasma cholesterol levels and development of atherosclerosis; epidemiological evidence that high dietary cholesterol was associated with high CVD incidence; and clinical studies showing that high cholesterol intake resulted in increased serum cholesterol levels. While the evidence of harm seems strong, each line of evidence was open to debate. Animal studies often used herbivores which were hypersensitive to dietary cholesterol as compared to omnivores (rabbit *versus* dog). Studies in suitable animal models often required use of pharmacological levels of cholesterol in the diet in order to elicit a response. Epidemiological data in the 1960s and 1970s relied on simple correlation analyses to show associations and did not account for collinearity of nutrients (saturated fat and cholesterol found in animal products). Eventually multivariate analysis of dietary lipids and CVD incidence documented that dietary cholesterol was not an independent risk factor [4,5,6]. Clinical feeding studies had two limits: use of pharmacological levels of dietary cholesterol (for example, six eggs per day for six weeks) and in the early studies reliance on measurement of total plasma cholesterol as the marker of risk [6]. Use of physiological dietary cholesterol levels and analysis of lipoprotein cholesterol distribution, including LDL size, provide a very different perspective on risk assessment [6].

Over the years the egg industry funded a variety of animal and clinical studies investigating the effects of egg intake on plasma total and lipoprotein cholesterol levels and metabolism under various conditions. These studies indicated that eggs had little effect on CVD risk [6]. Retrospective analysis of existing clinical data also indicated that eggs and dietary cholesterol, when consumed at physiological, not pharmacological levels, did not significantly affect CVD risk profiles [6,7,8]. Retrospective review of epidemiological studies which involved analysis of egg intake showed that egg intake was not associated with CVD incidence [6]. The more modern epidemiological studies using multivariate analysis reported that dietary cholesterol was non-significant as an independent factor in CVD incidence [4,5,6]. Based on these facts, the egg industry considered that it was justified in its efforts to challenge the egg restriction recommendation. Of course, one could predict a backlash to such a challenge.

## 3. Speaking with One Voice

By 1995 there was a concerted effort to unify all the US national dietary recommendations (so as not to confuse the public) such that the AHA guidelines, the NCEP guidelines, the Dietary Guidelines for Americans, and the Nutrition Facts Panel from the Food and Drug Administration (FDA) all set the dietary cholesterol recommendation at less than 300 mg/day (For detailed reviews of the history of the rationale and implementation of dietary lipid guidelines and their subsequent demise, see Taubes [7] and Teicholz [8].) One question rarely raised by these various agencies or by the scientific community was why 300 mg/day, why not 250 or 400 mg/day or whatever. Determining how the 300 mg/day number was chosen back in 1968 remains a mystery without a satisfactory answer other than it was half of what the estimated cholesterol intake was at the time. Irrespective of the scientific rationale for the number, it became established dogma in the nutrition community. Since one large egg contained 213 mg of cholesterol, per capita egg consumption continued to be low. ENC continued to argue against egg recommendations and presented the science documenting no significant effect of eggs on CVD risk. The counter argument was that the evidence showed that egg intake did increase plasma cholesterol levels [9,10], although at a level which was miniscule and offset by comparable increases in both LDL and HDL cholesterol levels with no change in the LDL:HDL ratio (*i.e.*, no change in relative risk) [11,12]. (1995 was also the year that I accepted the position of Executive Director of ENC).

## 4. A New Approach

In 1995 the egg industry initiated a two pronged approach to dealing with the dietary cholesterol issue: detailed studies of the effects of egg intake on CVD risk factors and studies of the contribution of eggs to a healthy diet across the lifespan. It was clear to the egg industry that simply funding more egg feeding studies was not going to significantly impact the perception of eggs as potentially harmful. Attempts to fund research analyzing data from large epidemiological studies were met with great resistance due to the curse of “industry funded research” negation and the hesitation of some researchers to be seen as not going along with the “consensus” that dietary cholesterol was harmful. And it was clear that no matter how many feeding studies were presented that the various agencies involved were not going to simply change the recommendations because they were wrong. In view of these factors the egg industry decided that it needed to give the policy makers a reason to change; *i.e.*, implement the “do no harm” imperative.

ENC initiated research projects on a wide variety of themes to document why eggs should be included in the diet. The eventual outcomes of these studies were a surprise to both the egg industry and health agencies. Many of these findings are reported in the associated papers in this issue of the journal.

1. Nutritional Value of Eggs: It is widely recognized that eggs are a highly nutritious food based on their high quality protein and compliment of vitamins and minerals [13]. Eggs are one of the most widely available economical sources of animal protein. In addition, many of the nutrients in eggs can be increased by altering the hen’s diet (Se, vitamin E, vitamin D, omega-3 fatty acids, xanthophylls and folate to name just a few). Due to the recommendation to eat no more than three whole eggs per week, and in some parts of Central and South America no more than two eggs per week, those in the lower socioeconomic classes have been scared away from an affordable source of high quality nutrition. It made little sense to restrict a highly nutritious food from the diets of those with sub-optimal nutrient intake whose main health issue was not the over consumption prevalent in the US. This became more obvious when considering the nutritional needs of growing children, pregnant women, and the elderly.
2. Egg Protein and Satiety: Egg protein, especially egg yolk protein, has a significantly greater satiety effect than other protein sources [14,15]. Studies have shown reduced caloric intake after an egg breakfast compared to a bagel breakfast [16], greater weight loss over 8 weeks with an egg as compared to a bagel breakfast as part of a hypocaloric diet [17], and larger changes in satiety hormones with an egg breakfast [18]. The five decade long shift from eggs for breakfast to carbohydrate rich cereals might not have been the best approach to weight maintenance and probably contributed to our national obesity problem.
3. Egg Protein and Sarcopenia: There are a number of factors which can impact the dietary availability of high quality animal protein for seniors: availability, affordability, preparation limits, and ease of chewing and digesting. Affordable sources of high-quality animal protein in the diet, especially eggs that are widely available and easy to cook, chew and digest, are of significant importance for growth and development in children as well as for reducing the rate of sarcopenia and maintaining lean muscle tissue mass in the elderly [19]. After 40 plus years of hearing about the dangers of egg cholesterol, many seniors studiously avoid eggs, probably to their detriment [20].
4. Egg Xanthophylls: Lutein and Zeaxanthin: Eggs provide highly bioavailable forms of the xanthophylls lutein and zeaxanthin which are related to lower risks for age-related macular degeneration and cataracts [21,22,23,24] as well as some types of cancer [22,25] and carotid artery atherosclerosis [26]. Studies showed that egg lutein had high bioavailability [21] and that adding eggs to the diet could result in significant increases in macular pigment optical density [23,24]. What continues to make this line of investigation so intriguing is that the levels of lutein and zeaxanthin in an egg can easily be increased up to ten-fold by adding marigold extract to the hens’ feed. In fact, today lutein enriched eggs are available in many parts of the world.
5. Egg Choline: Eggs are an excellent source of choline [27], an essential nutrient which has been shown to be inadequate in the diets of 9 out of 10 adults in the U.S. Choline plays an important role in fetal and neonatal brain development [28] and inadequate choline intake during pregnancy increases the risk for neural tube defects such as spina bifida [29]. Choline intake is also associated with decreased plasma levels of homocysteine and inflammatory factors, which are related to increased cardiovascular disease risk [30]. Recent studies have also shown that high intake of choline is associated with reduced breast cancer incidence and mortality [31,32]. Unfortunately, studies also show that a majority of the population, including a majority of pregnant and lactating women, do not have adequate choline intakes and that adding an egg a day to the diet could alleviate this inadequacy [33]. The importance of choline in fetal and neonatal brain development has been shown in numerous studies and inadequate choline intakes during these critical periods can have very negative effects [34,35,36].
6. The egg industry also supported a series of studies looking at the chronic and acute effects of egg intake on endothelial function in a variety of patients and found no evidence of adverse effects with daily egg ingestion on any cardiac risk factors in normolipidemic and hyperlipidemic adults or in adults with CVD [37,38,39].

## 5. An Outdated Hypothesis Slowly Put to Rest

In 1999 Hu *et al.* [40] published one of the first large, long term population studies on egg intake and CVD incidence. The results from over 117,000 men and women documented no differences in CVD risk between those consuming one egg a week *versus* one egg a day. Other large epidemiological studies have reported similar findings reporting that egg intake is not associated with CVD risk [41,42]. Meta-analyses have come to the same conclusion [43,44,45].

In 2002 the AHA dropped its specific egg restriction of 3–4 per week while keeping the less than 300 mg/day of dietary cholesterol guideline. While the US continued to recommend limits on dietary cholesterol, a large number of countries removed dietary cholesterol restrictions from their national dietary guidelines (Australia, Great Britain, Ireland to name a few). It took another twelve years for AHA to pronounce that “There is insufficient evidence to determine whether lowering dietary cholesterol reduces LDL-C.” [46]. In an equally surprising turn of events, the 2015 Dietary Guidelines Advisory Committee (DGAC) stated in its report that “Previously, the Dietary Guidelines for Americans recommended that cholesterol intake be limited to no more than 300 mg/day. The 2015 DGAC will not bring forward this recommendation because available evidence shows no appreciable relationship between consumption of dietary cholesterol and serum cholesterol, consistent with the conclusions of the AHA/ACC report.” [47]. A recommendation known worldwide that lasted for 47 years has simply been discarded and we can all go back to including eggs in our diets. The United States, which had first promoted the “no more than 300 mg/day cholesterol” recommendation, was now one of the last countries to do away with it. It is interesting to note that it required a much greater amount of research to prove that egg intake was unrelated to CVD risk than it did to initially condemn it as a significant contributor to CVD incidence. This nutritional saga shows that dietary recommendations need to be based more on science than belief and that industry funded research can be a valid approach to address specific issues and when applying proper scientific methods, can be of benefit to both the industry and the public.

## 6. Conclusions

For almost 50 years eggs and dietary cholesterol have been thought to contribute to high plasma cholesterol levels and increased CVD risk. Based on this belief dietary recommendations in the US and most countries have included dietary cholesterol and egg restrictions. Half a century of research have shown that egg and/or dietary cholesterol intake is not associated with increased CVD risk. In addition, research studies have shown that egg intake addresses a number of nutrient inadequacies and can make important contributions to overall health across the life span. Dietary cholesterol and egg restrictions have now been removed from most national dietary recommendations.
